# Role of C-Reactive Protein in Discrimination between Transudative and Exudative Pleural Effusions

**DOI:** 10.3390/diagnostics11112003

**Published:** 2021-10-28

**Authors:** Yana Kogan, Edmond Sabo, Majed Odeh

**Affiliations:** 1 Department of Internal Medicine A, Bnai Zion Medical Center, Haifa 3339419, Israel; janakgan37@gmail.com; 2 Pulmonary Division, Carmel Medical Center, Haifa 3436212, Israel; 3 Faculty of Medicine, Technion-Israel Institute of Technology, Haifa 3525422, Israel; EdmondSa@clalit.org.il; 4 Institute of Pathology, Carmel Medical Center, Haifa 3436212, Israel

**Keywords:** C-reactive protein, pleural effusion, transudate, exudate

## Abstract

Background: There is still no wide agreement regarding the efficacy of the serum levels of C-reactive protein (CRPs), pleural fluid levels of CRP (CRPpf), and their ratio (CRPr) in the discrimination between transudative (Tr) and exudative (Ex) pleural effusions (PEs). Most of the previous studies were conducted on small cohorts, and the role of CRPs in the CRPpf gradient (CRPg) in this discrimination has not been previously reported. The present study aims to assess the diagnostic efficacy of CRPs, CRPpf, CRPg, and CRPr in the discrimination between TrPE and ExPE in a relatively large cohort of patients with PE. Methods: The study population included 492 patients with PE, 210 of them with TrPE and 282 with ExPE. The levels of CRPs and CRPpf were measured, and the CRPg and CRPr were calculated. The values are presented as mean ± SD. Results: The mean levels of CRPs, CRPpf, CRPg, and CRPr of the TrPEs were 11.3 ± 5.7 mg/L, 4.6 ± 2.8 mg/L, 6.7 ± 3.9 mg/L, and 0.40 ± 0.14, respectively, and for the ExPEs, they were 140.5 ± 112.8 mg/L, 52.8 ± 53.2 mg/L, 87.2 ± 72.4 mg/L, and 0.37 ± 0.15, respectively. The levels of CRPs, CRPpf, and CRPg were significantly higher in the ExPEs than in the TrPEs (*p* < 0.0001). No significant difference was found between the two groups for the levels of CRPr (*p* = 0.15). The best cut-off value calculated by the receiver operating characteristic (ROC) analysis for discriminating TrPE from ExPE was for CRPs, 20.5 mg/L with area under the curve (AUC) = 97% and *p* < 0.0001; for CRPpf, 9.9 mg/L with AUC = 95% and *p* < 0.0001; and for CRPg, 13.6 mg/L with AUC = 96% and *p* < 0.0001. Conclusion: CRPs, CRPpf, and CRPg are strong markers for discrimination between TrPE and ExPE, while CRPr has no role in this discrimination.

## 1. Introduction

Pleural effusion (PE) is a common condition in clinical practice, and its prevalence is estimated to be about 400 cases/100,000 inhabitants [1,2]. The most common conditions that cause PE are congestive heart failure (CHF), pneumonia, and malignant neoplasms, but more than 50 causes can produce PE, including organic dysfunctions, lung or systemic diseases, diseases of the pleura itself, or problems due to drug treatments [3]. When a patient is found to have PE, an effort should be made to determine the cause. Distinguishing an exudate from a transudate is the first step in determining the cause of PE. Transudative PEs develop when there is a change in systemic factors, such as an increase in capillary hydrostatic pressure or a decrease in colloid osmotic pressure with no change in pleural surface. The leading causes of transudative PEs are CHF and cirrhosis. If the fluid is transudative, no further diagnostic procedures are necessary, and therapy is directed to the underline disease. Exudative PEs result from pleural inflammation, infection, injury, or lymphatic obstruction. The leading causes of exudative PEs are pneumonia, malignancy, viral infection, and pulmonary embolism [4,5]. Exudative PE always requires a more extensive and invasive diagnostic evaluation [4,5].

Traditionally, transudative and exudative PEs are distinguished depending on Light’s criteria [4,6]. PE is diagnosed as exudative if pleural fluid protein/serum protein > 0.5, and/or pleural fluid lactate dehydrogenase (LDH)/serum LDH > 0.6, and/or pleural fluid LDH is more than two-thirds the normal upper limit for serum, whereas transudative PEs meet none of these criteria. The main disadvantage of these criteria is that about 25% of PEs due to CHF are categorized according to Light’s criteria as exudates [4,7].

C-reactive protein (CRP) is an acute phase reactant synthesized and secreted in the liver by hepatocytes in response to various stimuli. The induction of CRP synthesis in the liver is triggered by pro-inflammatory mediators, such as interleukin-6 and tumor necrosis factor-α. The diagnostic value of the serum CRP (CRPs) level and pleural fluid CRP (CRPpf) level in the discrimination between transudative and exudative PEs is still controversial. Most of the previous studies demonstrated that CRPs, CRPpf, and the CRPpf/CRPs ratio (CRPr) are useful parameters for the discrimination between transudative and exudative PEs, but most of these studies were conducted on a small sample of patients, and their efficacy rate differs among these studies [8,9,10,11,12,13,14,15,16]. To date, the diagnostic value of CRPs in the CRPpf gradient (CRPg) in the discrimination between transudative and exudative PEs has not been assessed.

The aim of this retrospective study, which was conducted in a relatively large cohort of patients (492 patients) with PE, who were admitted to our Department of Internal Medicine at Bnai Zion Medical Center between January 2000 and October 2016, is to assess the diagnostic value of CRPs, CRPpf, CRPg, and CRPr in the discrimination between transudative and exudative pleural effusions.

## 2. Patients and Methods

### 2.1. Patients 

The study population consisted of 492 patients with PE who underwent thoracentesis and a full investigation regarding the cause of their PE with a final definitive diagnosis of the PE etiology. Of them, 210 patients, aged 33–96 years, had transudative PE, which was due to CHF in 200 patients, liver cirrhosis in 8 patients, and nephrotic syndrome in 2 patients. The rest of the 282 patients, aged 23–95 years, had exudative PE, which was due to pneumonia (parapneumonic) in 146 patients, malignancy in 126 patients, and tuberculosis in 10 patients. The PE was attributed to CHF when all of the following criteria were met: (1) cardiomegaly; (2) evidence of cardiac dysfunction (clinical, echocardiographic, and/or by MUGA scan); (3) radiological evidence of congested lungs and/or peripheral edema; and (4) response to treatment of CHF. In all cases, there was an absence of pulmonary embolism, purulent sputum, pulmonary infiltrates, and malignancy. PE was considered parapneumonic when it was associated with acute febrile illness with purulent sputum, pulmonary infiltrate, and responsiveness to antibiotic treatment in addition to chest tube drainage if the effusion was complicated. Effusion was considered malignant if malignant cells were demonstrated at cytological examination or in a biopsy specimen in the absence of other diseases causing PE. Tuberculous PE was diagnosed based on positive cultures for mycobacterium tuberculosis (of pleural fluid, sputum, or pleural biopsy specimens) or when the pleural biopsy specimen revealed typical epithelioid cell granulomas. PE was attributed to liver cirrhosis if a definitive diagnosis of cirrhosis was present in the absence of other causes of PE. PE was attributed to nephrotic syndrome based on a definitive diagnosis of nephrotic syndrome with the absence of other causes of PE. No PE was attributed to pulmonary embolism or connective tissue disease.

### 2.2. Methods

Data were collected from the patients’ charts. Only patients with a definitive diagnosis of the cause of their PE and with a measurement of CRPs and CRPpf were included in the study. PE was considered transudative if its cause was CHF, liver cirrhosis, or nephrotic syndrome, which were diagnosed according to the above-mentioned criteria. It was considered exudative if its cause was pneumonia, malignancy, or tuberculosis, which were diagnosed according to the above-mentioned criteria. CRPs and CRPpf levels were measured on a Cobas c 501 analyzer of Roche Diagnostics by C-Reactive Protein Gen.3 assay. The method is a particle-enhanced immunoturbidimetric, where human CRP agglutinates with latex particles coated with monoclonal anti-CRP antibodies. The aggregates are determined turbidimetrically at 546 nm, where the measuring range of the assay is between 0.3 and 350 mg/L, and the normal range is ≤5 mg/L. The study was conducted in accordance with the Declaration of Helsinki, and the protocol was approved by the Ethics Committee of Bnai Zion Medical Center. CRPg was calculated as CRPs–CRPpf, and CRPr was calculated as CRPpf/CRPs.

### 2.3. Statistical Analysis

Descriptive statistical values are presented as means ± standard deviation (SD) of means and 95% confidence intervals (CIs). The Kolmogorov–Smirnov test was carried out to evaluate the normality of the data. Comparisons between parametric groups were completed using the unpaired Student’s *t*-test. The *p*-values were corrected for multiple comparisons using Bonferroni correction. Receiver operating characteristic (ROC) analysis was used to detect the best cut-off values (i.e., those with the highest total accuracy) for separating transudative from exudative PEs. Sensitivity, specificity, positive predictive value (PPV), negative predictive value (NPV), total accuracy, odds ratio, and area under the ROC curves were calculated. The significance of the best cut-off values was evaluated using the χ^2^ test or the Fisher exact test as needed. Two-tailed *p*-values of ≤0.05 were considered statistically significant. 

## 3. Results

**Figure 1:**
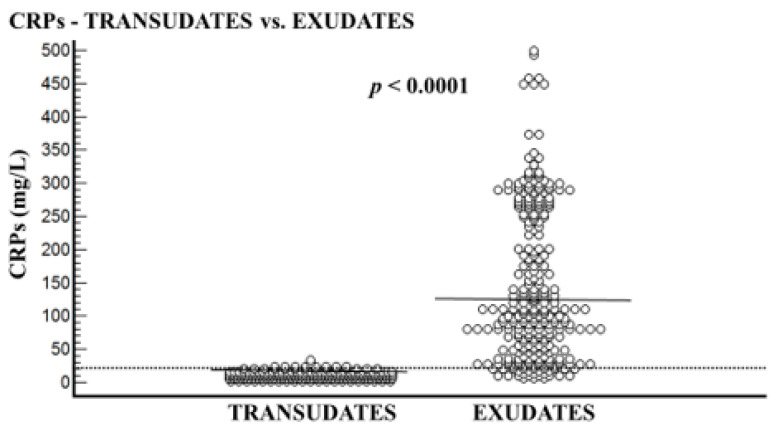
Serum CRP (CRPs) levels and their means for transudate group (11.3 mg/L) and exudate group (140.5 md/L), and best cut-off value (20.5 mg/L) for discrimination between the two groups. For both parameters *p* < 0.0001.

**Figure 2:**
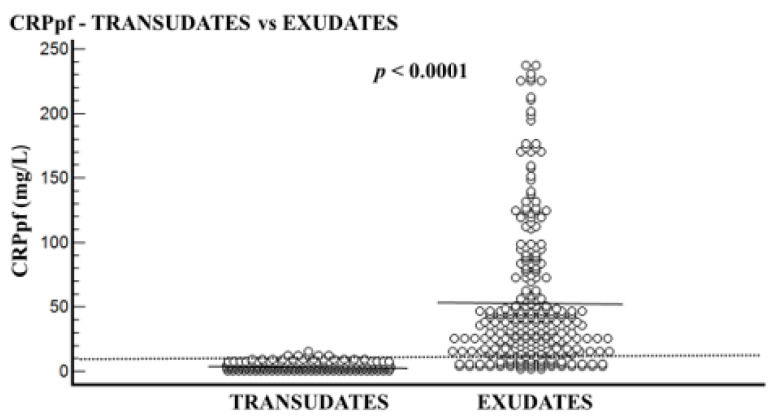
Pleural fluid CRP (CRPpf) levels and their means for transudate group (4.6 mg/L) and exudate group (52.8 mg/L), and best cut-off value (9.9 mg/L) for discrimination between the two groups. For both parameters *p* < 0.0001.

**Figure 3:**
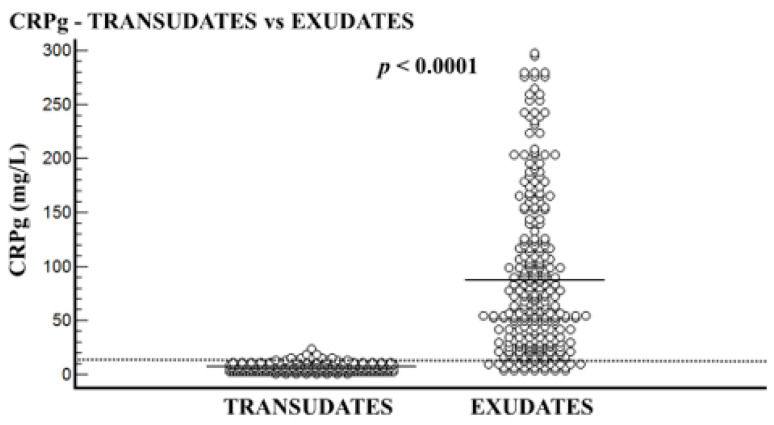
Serum and pleural fluid CRP gradient (CRPg) levels and their means for transudate group (6.7 mg/L) and exudate group (87.2 mg/L), and best cut-off value (13.6 mg/L) for discrimination between the two groups. For both parameters *p* < 0.0001.

**Figure 4:**
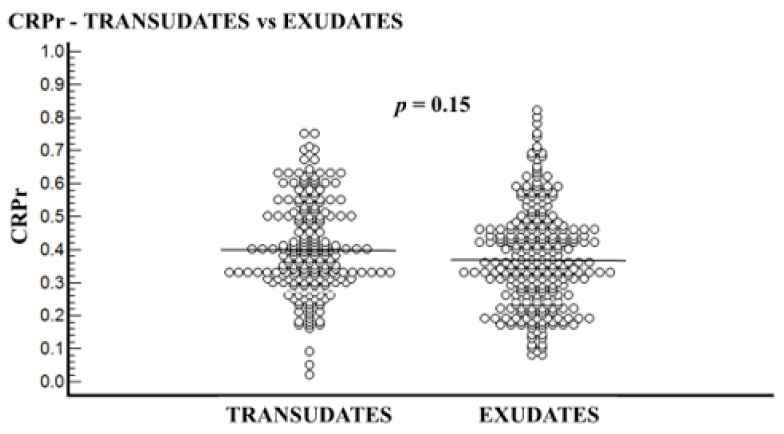
Pleural fluid to serum CRP ratio (CRPr) levels and their means for transudate group (4.0) and exudate group (0.37). No significant difference was found between the two groups (*p* = 0.15).

The transudative PE group included 210 patients, and the exudative PE group included 282 patients. In all patients, the CRPs level was higher than that of CRPpf. The mean age of the transudative group was significantly higher than the exudative group: 77.3 ± 10.4 years vs. 72.3 ± 14.6 years, respectively (*p* < 0.0001) (Table 1). The mean levels of CRPs, CRPpf, CRPg, and CRPr are presented in Table 1 and Figure 1, Figure 2, Figure 3 and Figure 4. The mean level of CRPs was significantly higher in the exudative group than in the transudative group: 140.5 ± 112.8 mg/L (95% CI: 127.3–153.7) vs. 11.3 ± 5.7 mg/L (95% CI: 10.5–12.1), respectively (*p* < 0.0001) (Table 1, Figure 1). The mean level of CRPpf was significantly higher in the exudative group than in the transudative group: 52.8 ± 53.2 mg/L (95% CI: 46.5–59.0) vs. 4.6 ± 2.8 mg/L (95% CI: 4.2–5.0), respectively (*p* < 0.0001) (Table 1, Figure 2). The mean level of CRPg was significantly higher in the exudative group than in the transudative group: 87.2 ± 72.4 mg/L (95% CI: 79.4–97.7) vs. 6.7 ± 3.9 mg/L (95% CI: 4.0–13.5), respectively (*p* < 0.0001) (Table 1, Figure 3). No significant difference was found between the exudative group and the transudative group for CRPr: 0.37 ± 0.15 (95% CI: 0.35–0.39) vs. 0.40 ± 0.14 (95% CI: 0.25–0.41), respectively (*p* = 0.15) (Table 1, Figure 4).

**Figure 5:**
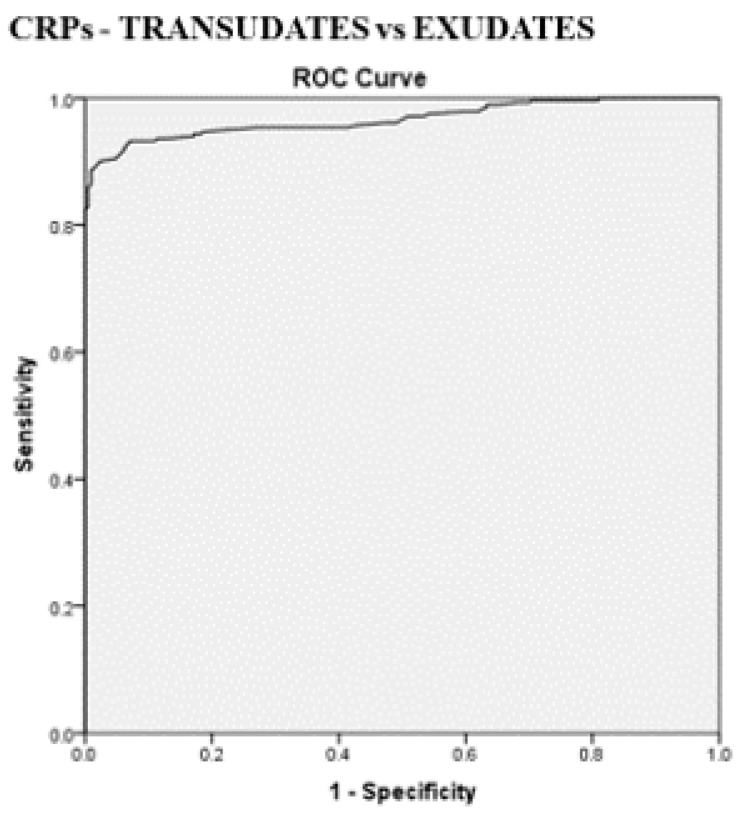
Receiver operating characteristic (ROC) curve of best cut-off value of serum CRP (CRPs), and its statistical characteristics, for discrimination between transudative pleural effusion and exudative pleural effusion. CRPs best cut-off value = 20.5 mg/L, AUC = 97% (95% CI: 95.2–98.2), sensitivity = 93.3%, specificity = 93.0%, total accuracy = 93.1%, PPV = 93.3%, NPV = 93%, odds ratio = 179.9 (risk for exudate when CRPs value > 20.5 mg/L), *p* < 0.0001.

**Figure 6:**
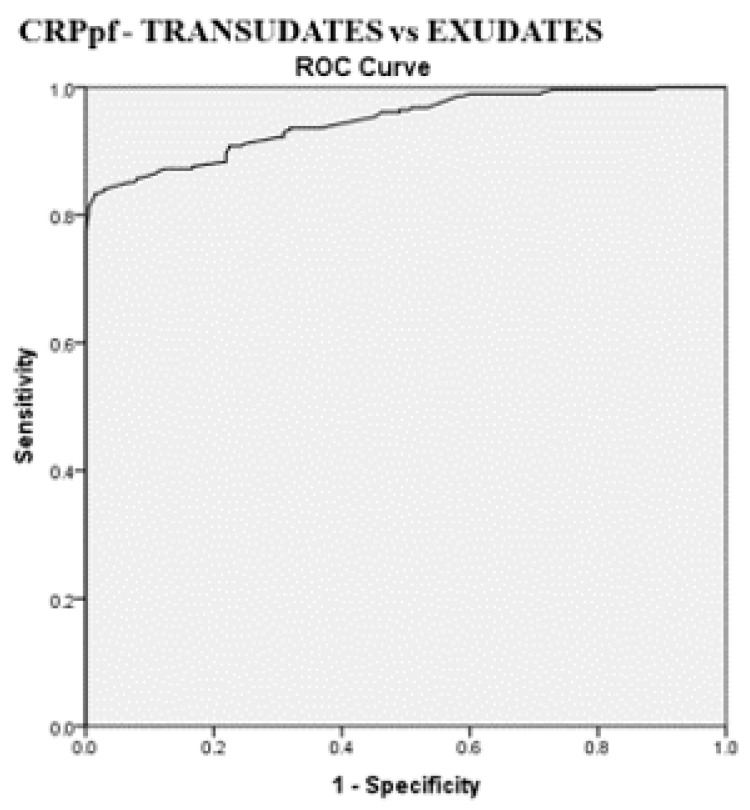
ROC curve of best cut-off value of pleural fluid CRP (CRPpf), and its statistical characteristics, for discrimination between transudative pleural effusion and exudative pleural effusion. CRPpf best cut-off value = 9.9 mg/L, AUC = 95% (95% CI: 92.9–96.5), sensitivity = 85.1%, specificity = 93.3%, total accuracy = 89%, PPV = 94%, NPV = 80%, odds ratio = 80 (risk for exudate when CRPpf value > 9.9 mg/L), *p* < 0.0001.

**Figure 7:**
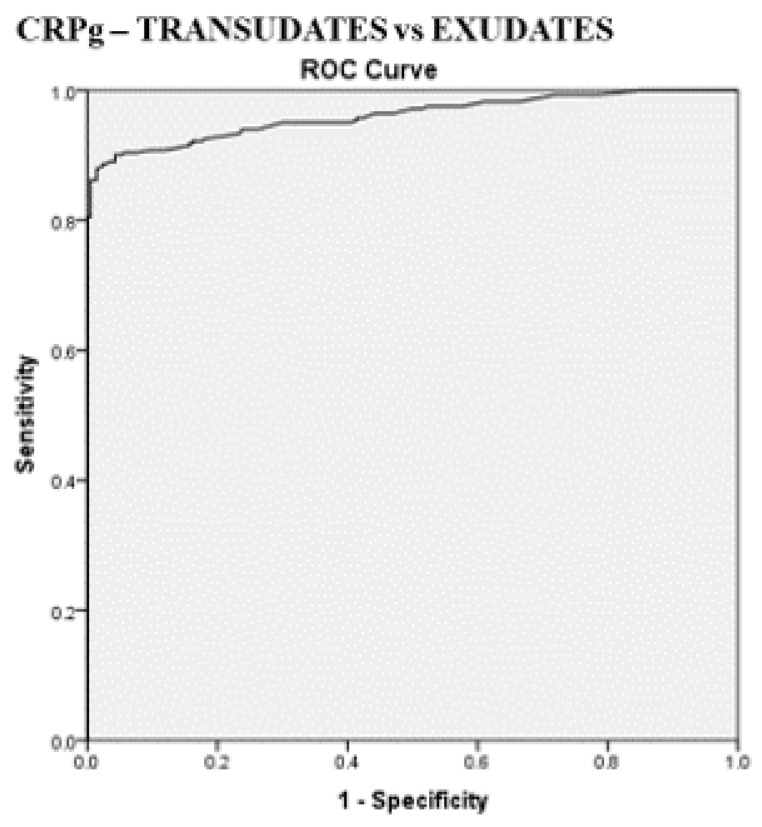
ROC curve of best cut-off value of serum and pleural fluid CRP gradient (CRPg), and its statistical characteristics, for discrimination between transudative pleural effusion and exudative pleural effusion. CRPg best cut-off value = 13.6 mg/L, AUC = 96.1% (95% CI: 94.4–97.7), sensitivity = 90%, specificity = 96%, total accuracy = 92%, PPV = 97%, NPV = 88%, odds ratio = 202.6 (risk for exudate when CRPg value > 13.6 mg/L), *p* < 0.0001.

The best cut-off values for CRPs, CRPpf, and CRPg, which were calculated by the ROC analysis for discriminating transudative from exudative PEs, together with their relevant statistical parameters, are presented in Figure 1, Figure 2 and Figure 3 and Figure 5, Figure 6 and Figure 7. 

The best cut-off value for CRPs was 20.5 mg/L with a sensitivity of 93.3%, specificity of 93%, total accuracy of 93.1%, AUC of 97%, and *p* < 0.0001 (Figure 1 and Figure 5). The best cut of value for CRPpf was 9.9 mg/L with a sensitivity of 85.1%, specificity of 93.3%, total accuracy of 89%, AUC of 95%, and *p* < 0.0001 (Figure 2 and Figure 6). The best cut-off value of CRPg was 13.6 mg/L with a sensitivity of 90%, specificity of 96%, total accuracy of 92%, AUC of 96.1%, and *p* < 0.0001 (Figure 3 and Figure 7).

## 4. Discussion

CRP is the classic acute phase reactant in inflammatory reactions, where its level in the blood increases due to various inflammatory processes of either infectious or noninfectious origin. It is synthesized primarily in the liver by hepatocytes, and its level in PE depends mainly on its level in the blood but also on the cause of PE. Thus, CRPpf levels are likely to reflect CRPs levels but cannot be equal or exceed CRPs levels as was found in all our patients.

The results of our study, which was conducted in a relatively large cohort of patients (492 patients), demonstrated that CRPs and CRPpf are both very useful markers for discrimination between transudative and exudative PEs, where CRPs is a little bit better than CRPpf in this discrimination. With a best cut-off value of 20.5 mg/L for CRPs, the discrimination between the two groups was excellent, where the AUC was 97% with a sensitivity of 93.3%, specificity 93%, total accuracy of 93.1%, and *p* < 0.0001. With a best cut-off value of 9.8 mg/L for CRPpf, the discrimination between the two groups was very good, where the AUC was 95% with a sensitivity of 85.1%, specificity of 93.3%, total accuracy of 89%, and *p* < 0.0001. These results are in agreement with the results of previous studies in this regard [8,10,11,13,14,15,16], but they are stronger and more accurate in indicating a strong role of CRPs and CRPpf in the discrimination between transudative and exudative PEs.

This is the first time that the role of CRPg in the discrimination between transudative and exudative PEs was investigated. The results of this study demonstrate that CRPg is also a very useful marker for discrimination between transudative and exudative PEs (Table 1, Figure 3 and Figure 7). With a best cut-off value of 13.6 mg/L for CRPg, the discrimination between the two groups was excellent, where the AUC was 96.1% with a sensitivity of 90%, specificity of 96%, total accuracy of 92%, and *p* < 0.0001 (Figure 7). These results indicate, for the first time, a strong role for CRPg in the discrimination between transudative and exudative PEs, even a little bit better than that of CRPpf.

Regarding the role of CRPr in the discrimination between transudative and exudative PEs, there were four published studies in this regard [9,12,13,16]. One study [13] with a level of CRPr < 1 in both groups, as was in our study, demonstrated no significant difference for CRPr between the two groups. The results of our study are in agreement with these results and oppose the results of the other three studies [9,12,16]. In one of these three studies [16], CRPr was <1 in both groups, where in the transudative group, which included only 67 patients (in our study, it included 210 patients), it was 0.36 ± 0.19, and in the exudative group, it was 0.64 ± 0.49, demonstrating a significant difference between the two groups (*p* = 0.0125). In one of the other two studies [9], CRPr was 2.8, and in the other [12], it was 1.1; these results are not reasonable because the level of CRPpf is always less than that of CRPs, as was found in all other studies, including ours. As mentioned above, CRP is produced in the liver, and its level in PE depends on its level in the blood and cannot exceed it or even equal it. The role of CRPr in the discrimination between transudative and exudative PEs should be further investigated.

This study has one limitation; it is a retrospective study.

## 5. Conclusions

The results of our study, which was conducted in a relatively large cohort of patients, demonstrated a very strong role for CRPs and CRPpf in the discrimination between transudative and exudative PEs, while CRPr had no significant role in this discrimination. It also demonstrated, for the first time, a very strong role of CRPg in this discrimination. According to these results, it can be concluded that if the PE is transudative, depending on its etiology, and the CRPs value is below 20.5 mg/L, diagnostic paracentesis is not needed unless it is also therapeutic. Further prospective studies on large cohorts of patients are needed in order to establish the strong role of CRPs, CRPpf, and CRPg in the discrimination between transudative and exudative PEs and in order to investigate the validity of CRPr in this discrimination.

## Figures and Tables

**Table 1:** Mean ± SD of age and mean ± SD and 95% CI of means of levels of CRPs, CRPpf, CRPg, and CRPr of transudate group and exudate group.

Parameter	Transudate (*n* = 210)	Exudate (*n* = 282)	*p*
Age (years)	77.3 ± 10.4	72.3 ± 14.6	<0.0001
CRPs (mg/L) 95% CI	11.3 ± 5.7 10.5–12.1	140.5 ± 112.8 127.3–153.7	<0.0001
CRPpf (mg/L) 95% CI	4.6 ± 2.8 4.2–5.0	52.8 ± 53.2 46.5–59.0	<0.0001
CRPg (mg/L) 95% CI	6.7 ± 3.9 4.0–13.5	87.2 ± 72.4 79.4–97.7	<0.0001
CRPr 95% CI	0.40 ± 0.14 0.25–0.41	0.37 ± 0.15 0.35–0.39	0.15

SD: standard deviation, CI: confidence interval, CRPs: serum C-reactive protein, CRPpf: pleural fluid CRP, CRPg: CRPs and CRPpf gradient, CRPr: CRPpf to CRPs ratio.
